# Quantification of Viral and Prokaryotic Production Rates in Benthic Ecosystems: A Methods Comparison

**DOI:** 10.3389/fmicb.2016.01501

**Published:** 2016-09-22

**Authors:** Eugenio Rastelli, Antonio Dell’Anno, Cinzia Corinaldesi, Mathias Middelboe, Rachel T. Noble, Roberto Danovaro

**Affiliations:** ^1^Department of Life and Environmental Sciences, Polytechnic University of MarcheAncona, Italy; ^2^Stazione Zoologica Anton Dohrn, NaplesItaly; ^3^Marine Biological Section, Department of Biology, University of CopenhagenHelsingør, Denmark; ^4^Institute of Marine Sciences, The University of North Carolina at Chapel Hill, Morehead CityNC, USA

**Keywords:** viral production, virus-induced prokaryotic mortality, epifluorescence microscopy, tritiated leucine, tritiated thymidine, deep-sea ecosystem, marine sediments

## Abstract

Viruses profoundly influence benthic marine ecosystems by infecting and subsequently killing their prokaryotic hosts, thereby impacting the cycling of carbon and nutrients. Previously conducted studies, based on different methodologies, have provided widely differing estimates of the relevance of viruses on benthic prokaryotes. There has been no attempt so far to compare these independent approaches, including contextual comparisons among different approaches for sample manipulation (i.e., dilution or not of the sediments during incubations), between methods based on epifluorescence microscopy (EFM) or radiotracers, and between the use of different radiotracers. Therefore, it has been difficult to identify the most suitable methodologies and protocols to be used as standard approaches for the quantification of viral infections of prokaryotes. Here, we compared for the first time different methods for determining viral and prokaryotic production rates in marine sediments collected at two benthic sites, differing in depth and environmental conditions. We used a highly replicated experimental design, testing the potential biases associated to the incubation of sediments as diluted or undiluted. In parallel, we also compared EFM counts with the ^3^H-thymidine incubations for the determination of viral production rates, and the use of ^3^H-thymidine versus ^3^H-leucine radiotracers for the determination of prokaryotic production. We show here that, independent from sediment dilution, EFM-based values of viral production ranged from 1.4 to 4.6 × 10^7^ viruses g^-1^ h^-1^, and were similar but overall less variable compared to those obtained by the ^3^H-thymidine method (0.3 to 9.0 × 10^7^ viruses g^-1^h^-1^). In addition, the prokaryotic production rates were not affected by sediment dilution, and the use of different radiotracers provided very consistent estimates (10.3–35.1 and 9.3–34.6 ngC g^-1^h^-1^ using the ^3^H-thymidine or ^3^H-leucine method, respectively). These results indicated that viral lysis was responsible for the abatement of 55–81% of the prokaryotic heterotrophic production, corroborating previous findings of the major role of viruses in benthic deep-sea ecosystems. Moreover, our methodological comparison for the analysis of viral production in marine sediments suggests that microscopy-based approaches are simpler and more cost-effective than those based on radiotracers. These approaches also reduce time to results and overcome issues related to generation of radioactive waste.

## Introduction

Viruses are key biological agents of prokaryotic mortality in the global oceans. By killing their hosts they play an important role in the functioning of the marine food webs and biogeochemical cycles (Weinbauer, 2004; Suttle, 2005, 2007). This also applies to benthic ecosystems where an important fraction of the prokaryotic C production can be transformed into organic detritus by viral lysis (Danovaro et al., 2008b). Accurate determinations of the quantitative role of viruses in the function of marine ecosystems, including their dramatic impacts on C and nutrient cycles, are crucially dependent on appropriate methods for assessing infection rates. There has been much debate on the accuracy and robustness of the different published approaches (Glud and Middelboe, 2004; Middelboe et al., 2006, 2011, Danovaro et al., 2008a; Siem-Jørgensen et al., 2008). However, to our knowledge, no highly replicated, formal comparison of methods has been published. Because of this, there has been little standardization across laboratories.

Most of the available methodologies that are currently utilized to determine viral production rates in benthic ecosystems are based on determinations of changes in viral abundances over time using time-course incubation experiments. These experiments have been previously conducted using: (a) homogenized and undiluted sediment samples (Glud and Middelboe, 2004; Middelboe and Glud, 2006; Middelboe et al., 2006); (b) undiluted and integer sediment samples (Siem-Jørgensen et al., 2008); and (c) sediments diluted with virus-free seawater (Mei and Danovaro, 2004; Danovaro et al., 2008a,b, 2009; Dell’Anno et al., 2009; Corinaldesi et al., 2012, 2010).

The incubation of undiluted sediments has been proposed to minimize the potential stimulation of microbial activity that might occur following sediment dilution, which could lead to an overestimation of viral production rates (Glud and Middelboe, 2004; Middelboe et al., 2006) and this approach has been applied to the analysis of viral infections in anoxic sediments. The incubation of sediments diluted with virus-free seawater has been derived from the method utilized for the water column (Wilhelm et al., 2002) and is useful to minimize the impact of protozoan grazing and to reduce the background concentration of viruses during incubation (Noble and Fuhrman, 1998; Noble and Steward, 2001). Other approaches have been utilized, such as transmission electron microscopy (TEM), which involves the viewing and counting of visibly infected cells (Filippini et al., 2006). However, these approaches are limited in their application due to the cost of the equipment necessary and the relative difficulty and level of technical training required to prepare and analyze a statistically robust number of replicate samples. Finally, incubations with radiolabeled substrates have the limit of relying on highly variable conversion factors (CFs) for estimating viral production from radioactive incorporation, as well as potential safety and waste disposal considerations (Steward et al., 1992, 1996; Noble and Steward, 2001; Helton et al., 2005; Danovaro et al., 2008b; Dell’Anno et al., 2009).

All of these different methodologies have been applied to a wide variety of benthic ecosystems (from coastal to deep-sea sediments under oxic or anoxic conditions, Glud and Middelboe, 2004; Mei and Danovaro, 2004; Middelboe and Glud, 2006; Middelboe et al., 2006; Danovaro et al., 2008b; Siem-Jørgensen et al., 2008, Corinaldesi et al., 2010, 2012, 2014). Independent evidence indicate that viruses are abundant and active in benthic ecosystems, with current measurements of viral abundance typically ranging from 10^8^ to 10^11^ virus g^-1^ of dry sediment and viral production rates in the order of 10^6^ to 10^8^ virus g^-1^ h^-1^ (Fischer et al., 2003; Hewson and Fuhrman, 2003; Middelboe et al., 2003; Glud and Middelboe, 2004; Mei and Danovaro, 2004; Danovaro et al., 2008a,b, 2015; Pinto et al., 2013). The same holds true for benthic prokaryotes, usually in the order of 10^7^ to 10^9^ cells g^-1^ of dry sediment and ranging in production rates from few nanograms to >1 μg of C g^-1^ h^-1^ (van Duyl and Kop, 1994; Dixon and Turley, 2001; Danovaro et al., 2008a,b). However, direct comparisons of values obtained across different studies has been hampered by the variety of methodological approaches used, as well as the array of environmental settings investigated.

Currently available estimates of viral production based on the dilution-based approach (Dell’Anno et al., 2009) are higher than those reported in different studies adopting undiluted sediment incubations (Danovaro et al., 2008a,b; Siem-Jørgensen et al., 2008; Pinto et al., 2013). Consequently, independent and contrasting evidence exists, suggesting low or high importance of viruses in the functioning of benthic ecosystems (Glud and Middelboe, 2004; Middelboe et al., 2006, 2011; Danovaro et al., 2008a,b). However, a synoptic comparison of the different technical procedures for sediment manipulation and of the laboratory analytical methods for the determination of viral and prokaryotic production rates is currently lacking, resulting in variable interpretation of the importance of viruses in certain systems.

In the present study, we compared for the first time different approaches for the analyses of viral and prokaryotic production rates to provide insights on the reliability of the most commonly used methodologies. To test for possible biases in the determinations based on different approaches of sediment manipulations, we used a highly replicated experimental design conducting parallel analyses of: (i) time-course incubations of intact sediment cores, (ii) incubations of homogenized and undiluted sediment samples, and (iii) incubations of sediments diluted 1-, 5-, or 10-times with virus-free seawater. Moreover, contextual comparisons were conducted to test the consistency between the determination of viral production rates by means of virus counting over time [using epifluorescence microscopy (EFM)] and by the method based on ^3^H-thymidine incorporation into viral genomes. Finally, the rates of prokaryotic production were determined by the use of different radiotracers (^3^H-thymidine or ^3^H-leucine) to provide independent assessment of the importance of viruses for prokaryotic mortality and production.

Results reported here will allow members of the research community to identify the advantages and limitations of the different methodologies, thereby promoting standardization and accuracy in the assessment of the vital roles that viruses play in benthic systems.

## Materials and Methods

### Study Areas and Sample Collection

Sediments were collected at two stations located at ca. 450 and 1900 m depth (42° 22.606 N, 03° 20.751 E, and 42°12.883 N, 04°15.429 E, respectively) in the NW Mediterranean Sea, by means of a NIOZ-type box-corer (0.25 m^2^ surface area; average sediment penetration depth of ∼40 cm), which allows collecting samples hermetically sealed. Three independent deployments were performed per station. Visual inspection of the overlying waters and sediment surfaces revealed very limited resuspension effects during sampling, consistent with previous studies on deep-sea sediments demonstrating no significant differences in microbiological variables analyzed synoptically on sediments collected with box-corers or multiple-corers (Danovaro et al., 1998). The replicate sediment cores used in the experiments described below were collected from each box core using sterile Plexiglas^®^ tubes. All incubations described in the following experiments were performed at *in situ* temperature (13–14°C) in the dark.

### Comparison of Different Approaches for Assessing the Effects of Sediment Manipulation on Viral and Prokaryotic C Production

In the present study, different approaches were used to identify possible biases induced by sediment manipulation on the determination of viral and prokaryotic heterotrophic C production rates. For the determination of viral production, we compared time-course experiments carried out on the top 1 cm sediment of: (i) intact and undiluted sediment cores (hereafter defined as whole core samples), (ii) homogenized and undiluted sediments (hereafter defined as undiluted samples), and (iii) sediments diluted 1, 5, or 10 times with virus-free seawater collected at the water-sediment interface of each station.

For the whole core experiment, a set of intact sediment cores were incubated and three independent replicates of their top 1 cm of sediment were collected at the beginning and after 3, 6, and 12 h of incubation. A second set of cores was used for the parallel time-course incubation experiments conducted on undiluted and on diluted sediments, keeping the same time intervals. In these experiments, the top 1 cm of sediment subsamples were transferred in sterile Whirl-pak^®^ bags and homogenized (for the undiluted samples), or transferred to sterile plastic jars and diluted 1, 5, or 10 times with virus-free seawater previously collected at the water-sediment interface (for the diluted samples).

For the determination of prokaryotic heterotrophic C production, parallel time-course experiments were carried out both on whole core samples and on the diluted top 1 cm of sediments, as detailed below.

### Determination of Viral Production from Epifluorescence Microscopy EFM Counts

The samples collected at each time interval from the different treatments were analyzed for viral abundance by EFM after the extraction of viruses from the sediments using pyrophosphate (final concentration, 5 mM) and ultrasound treatment (three 1-min treatments using a Branson Sonifier 2200; 60W) (Danovaro et al., 2001; Danovaro and Middelboe, 2010). Samples were then diluted from 100- to 500-fold with sterile and virus-free water (filtered through 0.02-μm-pore-size filters), treated with DNases (to remove extracellular DNA) and filtered onto 0.02 μm pore size filters (Anodisc Al_2_O_3_, 25 mm diameter). The filters were stained using SYBR Green I (10000× in anhydrous dimethyl sulfoxide, Molecular Probes-Invitrogen), incubated in the dark for 20 min and mounted on glass slides with a drop of 50% phosphate buffer (6.7 mmol L^-1^; pH 7.8) and 50% glycerol containing 0.5% ascorbic acid (Noble and Fuhrman, 1998). Viral counts were performed under EFM (magnification, ×1000; Zeiss filter set #09, 488009-9901-000, excitation BP 450–490 nm, beam splitter FT 515, emission LP 520), by examining at least 20 fields per slide and counting at least 400 viral particles per filter. The viral production rates were determined from linear regression analyses of the increase of viral abundances versus time (Dell’Anno et al., 2009), and data were normalized to sediment dry weight after desiccation (48 h at 60°C).

### Determination of Viral Production from ^3^H-Thymidine Incorporation

The viral production rates determined by EFM were compared with those obtained by incorporation experiments of ^3^H-thymidine into viral genomes, using a modification of the methodology previously conducted in seawater (Steward et al., 1992; Fuhrman and Noble, 1995). Replicate sediment samples (*n* = 3) of the top 1 cm were diluted 1:1 with 0.2-μm-pre-filtered seawater (collected at the sediment water interface of each station) containing ^3^H-thymidine (specific activity 86 Ci mmol^-1^, final concentration 0.2 μM) and gently mixed. Parallel time-course experiments of concentration-dependent incorporation (from 0.05 to 5.0 μM ^3^H-thymidine) indicated substrate saturation during incubations. Ten ml aliquots were collected at time 0 and after 3, 6, and 12 h and stored at -20°C until further processing. For extraction of viruses, samples were treated using pyrophosphate and ultrasound (see above) to detach viral particles. Samples were then centrifuged (3000 × *g* 10 min) and the supernatant fluids were immediately transferred to sterile 15 ml tubes. The sediment pellets were then subjected to two additional washes with 5 ml of pre-filtered virus-free seawater and centrifugation (3000 × *g*; for 10 min). The supernatants were combined and filtered through 0.2 μm pore size polycarbonate filters (Nuclepore) to remove residual sediment particles and prokaryotic cells. An aliquot of the filtered supernatant was used for viral and prokaryotic counts by EFM as described above, to confirm the removal of prokaryotic cells from the filtered samples. The remaining supernatants were then divided into two equal aliquots, treated with DNase I and RNase (final concentration 5 U ml^-1^ each) to remove extracellular nucleic acids, and incubated at room temperature for 1 h. After incubation, the enzymes were inactivated by adding formalin (2% final concentration) and samples were chilled on ice for 10 min. A carrier solution containing DNA, RNA and bovine serum albumine (each at 50 μg ml^-1^) was then added to each aliquot of sample. One aliquot was treated with cold trichloroacetic acid (TCA, 5% final concentration) and incubated for 1 h on ice (hereafter defined “cold sample”), whereas the other, after TCA addition, was incubated for 1 h at 100°C (hereafter defined “hot sample”). After incubation, all samples were vigorously shaken, filtered through 0.2 μm pore size polycarbonate filters (Nuclepore), then incubated for 1 h at 100°C with HCl 1N. The radioactivity in the sample was then measured by liquid scintillation counting (Packard Tri-Carb, 2100). The moles of thymidine incorporated into viruses (TdR_inc_) per g dry sediment (60°C, 24 h) per hour were obtained using the following formula:

$${TdR}_{inc}=({DPM}_{inc})/\left[(S.A.\times 2.22\times 10^{12}{DPMCi}^{-1})\times g\times h\right]$$

where: DPM_inc_ is the difference between the disintegration per minute (DPM) in the cold and in the hot sample; S.A. is the specific activity of the ^3^H-thymidine (Ci mol^-1^); g is the sediment dry weight in grams; h is the incubation time (hours).

Differences of the radioactivity between “cold and hot samples” obtained at the beginning of incubation (time 0) were negligible (close to 0), whereas the radioactivity in cold samples was always significantly higher than in hot samples after 3, 6, and 12 h of incubation (up to ca. 20 times higher).

In the present study, sample-specific CFs were experimentally determined, according to Steward et al. (1992), as the inverse of the slope of the linear regression between the moles of ^3^H-thymidine incorporated g^-1^ vs. viruses g^-1^ determined on the same sample by EFM. The moles of ^3^H-thymidine incorporated per unit of time were then converted into estimates of viral production rates (i.e., viruses g^-1^ h^-1^) on the basis of these sample-specific CFs, and compared with those resulting from the use of the CFs previously published. These included the theoretical CF of 0.024 × 10^21^ viruses per mole of ^3^H-thymidine incorporated (Noble and Steward, 2001), and those empirically determined in previous studies, of 0.175 × 10^21^ (Danovaro et al., 2008b), 0.617 × 10^21^ (Steward et al., 1992), and 2.1 × 10^21^ (Steward et al., 1992) viruses produced per mole of ^3^H-thymidine incorporated.

### Prokaryotic Abundance and Biomass

The prokaryotic abundance in the deep-sea sediments was determined from the same sediment samples used for the viral counts. The prokaryotic cells were extracted from the sediments according to standard procedures, stained with SYBR Green I, and counted under EFM (Danovaro et al., 2008a). For the determination of the prokaryotic biomass, the cell biovolume obtained from prokaryotic size following inter-calibration with scanning electron microscopy based size determinations was converted into carbon content assuming 310 fg C μm^-3^ (Fry, 1990) in line with previous studies (Danovaro, 2010 and references therein; Danovaro et al., 2015). Prokaryotic biomass per cell was calculated as total biomass divided by total prokaryotic abundance.

### Determination of Prokaryotic C Production Using ^3^H-Leucine

For the determination of prokaryotic C production, the top 1 cm of intact sediment cores was spiked with ^3^H-leucine (specific activity, 68 Ci mmol^-1^) previously diluted in virus-free seawater collected from the water-sediment interface. A final concentration of 0.2 μM was reached in the top 1 cm sediment layer by adding 2 μl aliquots of ^3^H-leucine solution on the top and at 0.5 cm below the sediment surface, homogeneously covering the whole core area. In parallel, subsamples of the diluted sediment slurries (described above) were incubated with ^3^H-leucine (at the same final concentration) and sampled for measurement of ^3^H incorporation into prokaryotic biomass. The whole cores and the diluted sediment samples where then incubated up to 6 h to check for the linearity in the incorporation of radiolabeled substrate. Parallel time-course experiments of concentration-dependent incorporation (from 0.05 to 5.0 μM ^3^H-leucine) indicated substrate saturation during incubations. Blanks for each sediment sample were added with ethanol (80%) immediately before ^3^H-leucine addition. After incubation, samples were supplemented with ethanol (80%), centrifuged, washed again two times with ethanol (80%), and the sediment was finally re-suspended in ethanol (80%) and filtered onto polycarbonate filters (0.2 μm pore size; vacuum <100 mm Hg). Subsequently, each filter was washed four times with 2 ml of 5% TCA, then transferred into a Pyrex tube containing 2 ml of NaOH (2M) and incubated for 2 h at 100°C. After centrifugation at 800 × *g*, 1 ml of supernatant fluid was transferred to vials containing an appropriate scintillation liquid. The incorporated radioactivity in the sediment samples was measured with a liquid scintillation counter (Packard Tri-Carb, 2100). The prokaryotic heterotrophic C production was calculated as follows:

$$\mathrm{Pr}okaryotic\,\;heterotrophic\,\;C\,\;production=LI\times 131.2\times {\left(\%Leu\right)}^{-1}\times (C/protein)\times ID$$

where: LI is the leucine incorporation rate (mol g^-1^ h^-1^), 131.2 is the molecular weight of leucine, %Leu is the fraction of leucine in a protein (0.073), C/protein is the ratio of cellular carbon to protein (0.86; Simon and Azam, 1989), and ID is the isotope dilution, assuming a value of 2.

### Determination of Prokaryotic C Production Using ^3^H-Thymidine

To test for the consistency of the determination of the prokaryotic C production using ^3^H-leucine, a second set of intact whole cores and of diluted sediment samples was added with ^3^H-thymidine at substrate-saturation concentration (specific activity 86 Ci mmol^-1^, final concentration 0.2 μM; van Duyl and Kop, 1990). The sediment samples were then incubated in parallel with those treated with ^3^H-leucine, and at the same time intervals incubations were stopped with ethanol (80%), samples were centrifuged and washed again two times with ethanol (80%), and the supernatants filtered onto 0.2 μm pore size polycarbonate filters. The filters were transferred into pyrex tubes, added with 5% TCA and heated for 30 min at 100°C before liquid scintillation countings. The non-specific binding of ^3^H-thymidine to the sediments was taken into account by analyzing replicate sediment sub-samples treated with 80% ethanol before ^3^H-thymidine addition (i.e., sediment blanks). Prokaryotic C production was calculated assuming the CF previously reported for deep-sea sediments of 2 × 10^18^ cells produced per mole thymidine incorporated and on the basis of the C content of prokaryotic cells (Dixon and Turley, 2001).

### Burst Size and Virus-Induced Prokaryotic Mortality

Prokaryotic burst size (BS, i.e, the number of viruses released by each cell lysed due to viral infection) was estimated from time-course experiments of viral production following Mei and Danovaro (2004), and using the equation:

$$BS=VP/P_{killed}$$

where: VP is the number of viruses produced g^-1^ h^-1^, determined as described above for the assessment of viral production rates by EFM, while P_killed_ is the number of prokaryotic cells killed g^-1^ h^-1^, estimated as follows:

$$P_{killed}=\left(P_{start}+P_{prod}\right)-\left(P_{end}\right)$$

where: P_start_ is the prokaryotic abundance at start of incubations as determined by EFM (see “Materials and Methods” above); P_prod_ is the number of prokaryotic cells produced in the interval of incubation calculated as prokaryotic C production (determined by the radiotracer incubation experiments as described above) divided by prokaryotic biomass per cell (see methods above for details on biomass estimates); and P_end_ is the number of prokaryotes actually counted after the incubation interval by EFM (Mei and Danovaro, 2004; Danovaro et al., 2008a).

The virus-induced prokaryotic mortality was calculated following Danovaro et al. (2008b) as:

$$\left(P_{killed}/P_{prod}\right)\times 100$$

i.e., dividing the number of cells killed by viruses g^-1^ h^-1^ by the total number of prokaryotes produced g^-1^ h^-1^, and multiplying by 100 to express the value as percentage.

### Statistical Analyses

The differences in viral abundance over time (i.e., during the 12 h incubations) were tested by one-way analysis of variance (ANOVA) followed by pair-wise test when significant differences were encountered. To test for differences in the viral production rates obtained from time-course experiments carried out on intact and undiluted sediment samples, homogenized and undiluted sediments, and diluted sediments, analysis of variance was carried out. Analysis of variance was also carried out to test for differences in the viral production rates and virus-induced prokaryotic mortality values obtained using EFM and those determined by ^3^H-thymidine incorporation experiments as well as to test for differences between prokaryotic C production rates obtained using ^3^H-thymidine and ^3^H-leucine. Before analysis, the homogeneity of variance was checked using the Cochran’s test on appropriately transformed data. Analysis of covariance (ANCOVA) was conducted to test the differences in the rates determined from the regression analysis of viral counts by EFM over time and of ^3^H-thymidine incorporation over time.

## Results

### EFM Analyses and Effects of Sediment Dilution on Viral Production Rates

Viral abundances were significantly lower in the sediments collected at 450 m depth than at 1900 m depth (on average 3.0 ± 0.3 × 10^8^ viruses g^-1^ as compared to 7.1 ± 0.3 × 10^8^ viruses g^-1^, respectively; ANOVA, *p* < 0.01). Similarly, benthic prokaryotic abundance and biomass were significantly lower at the shallow station (1.8 ± 0.1 × 10^8^ cells g^-1^, corresponding to an average 4.6 μg C g^-1^) than at the deeper one (3.6 ± 0.1 × 10^8^ cells g^-1^, corresponding to an average 7.9 μg C g^-1^; ANOVA, *p* < 0.01).

**FIGURE 1:**
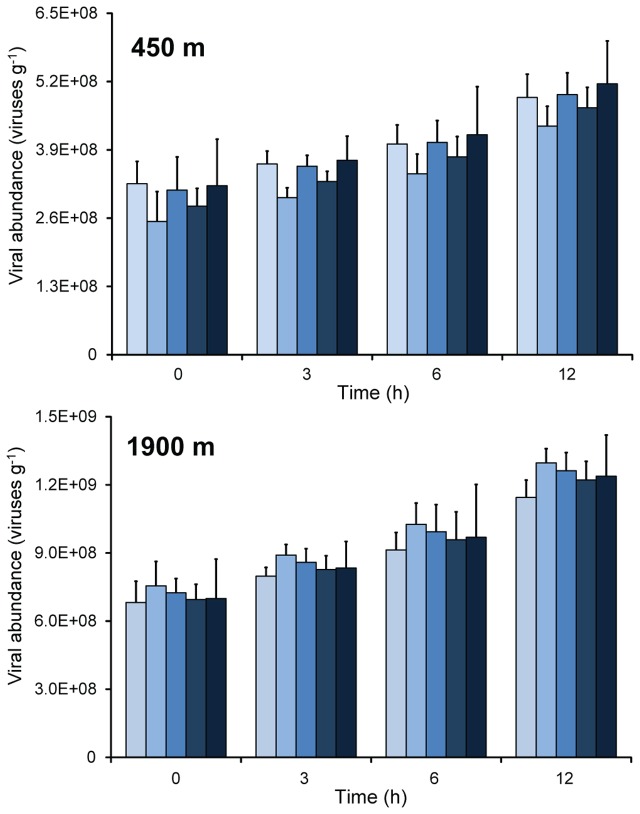
**Changes of viral abundance during time course incubation experiments carried out on sediment samples collected at the two benthic deep-sea sites (at 450 and 1900 m water depth).** The bars refer to, from light to dark blue: sediments diluted 1:1, 1:5, and 1:10 times; homogenized and undiluted; intact sediment cores. Mean values (*n* = 3) and SDs are reported.

At both stations, there was a linear increase in viral abundance during the time-course experiments carried out on intact undiluted sediment samples, on homogenized undiluted sediments, and on sediments diluted 1, 5, or 10 times (**Figure 1**). Statistical analyses confirmed that in all samples viral abundances increased significantly from the beginning of the experiments to 6–12 h (ANOVA, *p* < 0.01).

**FIGURE 2:**
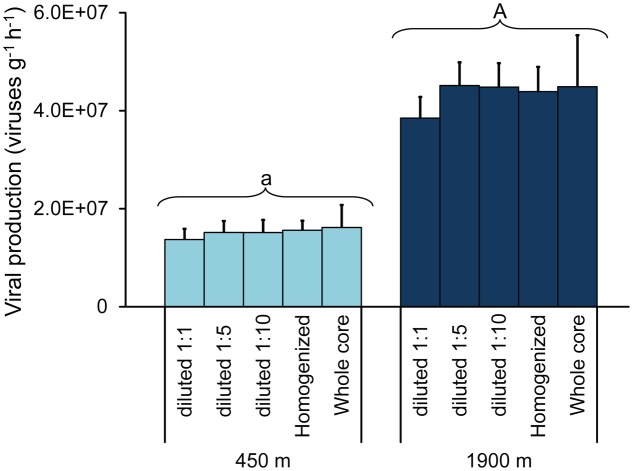
**Viral production rates obtained on diluted sediments (1:1, 1:5, and 1:10 times); homogenized and undiluted sediments and intact sediment cores collected at 450 m (light blue bars) and at 1900 m water depth (dark blue bars).** Mean values (*n* = 3) and SDs are reported. The upper case letter “A” for the station at 1900 m indicates values significantly higher (*p* < 0.01) than at 450 m water depth (marked with lower case letter “a”).

The increase in viral abundances over time was similar in all incubations, with no significant differences in viral production between the different sediment manipulation approaches used (**Figure 2**; ANCOVA, n.s.). Viral production rates obtained from whole-core incubation experiments displayed wider variability (coefficient of variation: 26 ± 3%) when compared with values of the incubations based on homogenization or dilution of the sediment samples (coefficient of variation: 14 ± 3%). Overall, values of viral production obtained through the EFM-based approach, independent of the approach used for sediment manipulation, were significantly higher in the sediments of the deeper station compared to the shallow one [4.3 ± 0.6 × 10^7^ viruses g^-1^h^-1^ and 1.5 ± 0.3 × 10^7^ viruses g^-1^h^-1^, respectively; (**Figure 2**; ANCOVA, *p* < 0.01)].

### Comparison of Viral Production Rates Obtained by EFM and by the ^3^H-Thymidine Method

**FIGURE 3:**
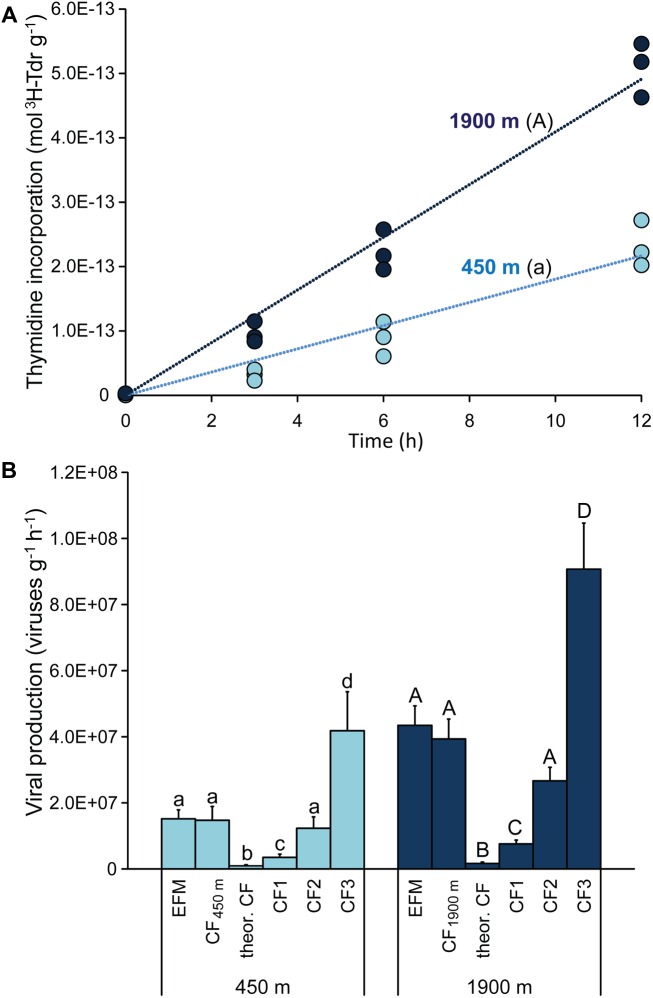
**(A)** Shows values of incorporation of tritiated thymidine into viral DNA obtained during time course incubation experiments carried out on sediment samples collected at the two benthic deep-sea sites. The upper case letter “A” for the station at 1900 m indicates that the incorporation rate of ^3^H-thymidine was significantly higher (*p* < 0.01) than at 450 m water depth (marked with lower case letter “a”). **(B)** Reports the comparison of viral production rates obtained by the EFM method with those estimated based on the ^3^H-thymidine method using different CFs. These include: (i) the sample-specific CFs determined for the two stations analyzed in the present study (CF_450m_, and CF_1900m_) of, respectively, 0.74 × 10^21^ and 0.91 × 10^21^ viruses produced per mole of ^3^H-thymdine incorporated; (ii) the theoretical CF of 0.024 × 10^21^ viruses mol^-1^ (Noble and Steward, 2001); and (iii) the CFs empirically determined in previous studies (CF1, CF2, and CF3) of, respectively, 0.175 × 10^21^ (Danovaro et al., 2008b), 0.617 × 10^21^ (Steward et al., 1992), and 2.1 × 10^21^ (Steward et al., 1992) viruses mol^-1^. Different letters indicate significant differences (*p* < 0.01) among the values obtained using different CFs, and upper case letters for the station at 1900 m water depth indicate significant differences (*p* < 0.01) compared with the corresponding values reported for the station at 450 m water depth.

Viral production experiments carried out using the ^3^H-thymidine method revealed a significant (*p* < 0.01) and linear increase of the ^3^H-thymidine incorporated over time (**Figure 3A**). ANCOVA analyses indicated that the rate of incorporation of ^3^H-thymidine was significantly higher for sediments collected at 1900 m (**Figure 3A**; *p* < 0.01). The experimentally determined CFs obtained in our study by calculating the inverse of the slope of the regression line of moles of ^3^H-thymdine incorporated g^-1^ versus direct counts of viruses g^-1^ (according to Steward et al., 1992) were 0.74 and 0.91 × 10^21^ viruses produced per mole of ^3^H-thymdine incorporated at 450 and 1900 m depth, respectively. The application of these sample-specific CFs resulted in viral production rates very similar to those found by EFM (1.5 ± 0.4 and 4.0 ± 0.6 × 10^7^ viruses g^-1^ h^-1^ at 450 and 1900 m depth, respectively; **Figure 3B**). Conversely, the application of the theoretical CF reported by Noble and Steward (2001) resulted in the lowest virus production rates (0.5 ± 0.1 and 1.1 ± 0.2 × 10^6^ viruses g^-1^ h^-1^ at 450 and 1900 m depth; **Figure 3B**). The assumption of the different empirical CFs published so far provided viral production rates ranging from 0.32 to 4.2 × 10^7^ viruses g^-1^ h^-1^ for sediments at 450 m, and from 0.71 to 9.0 × 10^7^ viruses g^-1^ h^-1^ at 1900 m depth. In general, the assumption of a constant CF for the two different deep-sea stations confirmed that the viral production rates were significantly higher in the sediments collected at 1900 m than at 450 m (ANOVA, *p* < 0.01), in line with what was evidenced by the EFM approach (**Figure 3B**). However, assuming different CFs in the radiotracer method resulted in a wide range of possible values of viral production, with very high overall coefficient of variation of 105–130% (compared with the coefficient of variation of 14–20% of the EFM method).

### Prokaryotic C Production Rates Using the ^3^H-Thymidine or ^3^H-Thymidine Method on Diluted and Undiluted Sediment Samples

**FIGURE 4:**
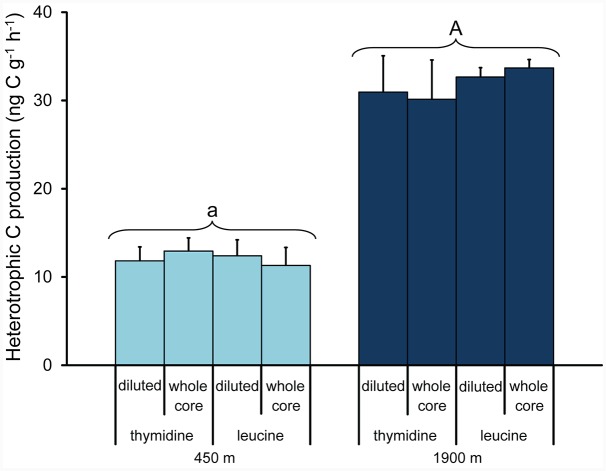
**Comparison between prokaryotic heterotrophic carbon production determined by parallel incorporation experiments of ^3^H-thymidine or ^3^H-leucine carried out on diluted sediments (1:1) and intact sediment cores collected at the two benthic deep-sea sites (at 450 and 1900 m water depth).** Mean values (*n* = 3) and SDs are reported. The upper case letter “A” for the station at 1900 m indicates values significantly higher (*p* < 0.01) than at 450 m water depth (marked with lower case letter “a”).

The two methods used to determine prokaryotic heterotrophic carbon production rates (i.e., the ^3^H-thymidine and ^3^H-leucine protocols) produced very similar results, independent of the approach used (i.e., diluting the samples vs whole sediment core incubations, **Figure 4**; ANOVA, n.s.). Prokaryotic heterotrophic carbon production rates were significantly higher in the sediment collected at 1900 m depth than at 450 m depth (on average, 32 ± 3 ng C g^-1^ h^-1^ and 12 ± 2 ng C g^-1^ h^-1^, respectively; ANOVA, *p* < 0.01), resulting in prokaryotic turnover times significantly faster at the deeper station (on average, 10.6 ± 0.9 days vs. 15.0 ± 2.2 days at 1900 and 450 m depth, respectively).

### Assessment of the Impact of Viruses on Their Prokaryotic Hosts

The impact of viruses on prokaryotes in the analyzed sediments was expressed as virus-induced prokaryotic mortality, calculated as the percentage of prokaryotes lysed by viruses relative to the number of prokaryotes produced within the same incubation interval (as determined by the radiotracer incubation experiments). In this study, the empirically determined values of burst size (needed to calculate the number of prokaryotic cells lysed by viruses and calculated based on average values of viral production obtained by EFM, see “Materials and Methods” for details) resulted in average 54 and 37 viruses produced per lysed cell at 450 and 1900 m depth, respectively. Based on these values, viruses were estimated to be responsible for the abatement of 55–62% and 75–81% of the prokaryotes produced in the sediment at 450 and 1900 m depths, respectively. Using the average values of viral production determined by the ^3^H-thymidine method (i.e., based on the assumption of the average CF, **Figure 3B**) resulted in values of virus-induced prokaryotic mortality not statistically different from those determined by EFM (ANOVA, ns.).

## Discussion

### Different Sediment Processing Approaches for Assessing Viral Production Rates

Several approaches for quantification of viral production in marine sediments have been applied in different studies and their advantages and limitations discussed. For instance, the incubations of undisturbed and/or undiluted sediments have the advantage of minimum disturbance of the sediment (Siem-Jørgensen et al., 2008), but they do not take into consideration the loss of viruses due to benthic grazers, enzymatic degradation processes (Dell’Anno et al., 2015), or the impact of new viral infections during incubation (Danovaro et al., 2008a). The use of sediment-dilution approaches, on the other hand, alter the physical and chemical properties of the sediment, potentially stimulating prokaryotic activity (Hansen et al., 2000), as well as possibly inducing lysogenic cells, both of which would contribute to increases in viral production. At the same time, the dilution approach reduces viral and host densities, allowing determination of even small increases in viral abundance and minimizing the effects of reinfection during the incubations. On one hand, sediment dilution makes the effect of protozoa and other potential predators on benthic viruses negligible, and it can decrease the concentration of extracellular enzymes, potentially reducing viral loss due to enzymatic degradation. On the other, in undisturbed sediments, viruses attached to particles may be partially protected from enzymatic degradation, whereas possible detachment into the water due to sediment manipulation and/or dilution would expose the whole viral surface to enzymatic degradation, potentially increasing the rates of viral loss.

Available estimates of viral production rates determined by incubation experiments carried out on diluted sediment samples (i.e., using the dilution-based approach; Dell’Anno et al., 2009) are generally higher than those reported by studies adopting the incubation of homogenized and undiluted sediments (Danovaro et al., 2008a,b; Siem-Jørgensen et al., 2008; Pinto et al., 2013). For this reason, it has been hypothesized that elevated rates of viral production may result from a stimulation of prokaryotic metabolism following sediment manipulation (Glud and Middelboe, 2004; Middelboe et al., 2006, 2011).

The present study represents the first attempt to systematically investigate the influence of the different approaches adopted for sediment manipulation and of the different analytical laboratory methodologies used for the assessment of viral production rates. The results from our experiments, replicated in two different benthic environments, reveal that the different approaches tested for sediment manipulation did not significantly affect viral production rates. This suggests that previous estimates obtained using the dilution-based approach (e.g., Danovaro et al., 2008b; Dell’Anno et al., 2015) are not biased by sample processing (i.e., sediment dilution). Our study also reveals that the dilution-based approach is not only accurate for the determination of benthic viral production rates, but it also provides rate determinations that have lower variability than those obtained through the incubation of undisturbed undiluted sediments. These results allow concluding that the very different viral production rates observed so far across different studies (that have resulted in highly contrasting interpretations of the ecological and biogeochemical roles of benthic viruses; see e.g., Hewson and Fuhrman, 2003; Middelboe et al., 2006; Danovaro et al., 2008b; Corinaldesi et al., 2010, 2012; Middelboe et al., 2011; Dell’Anno et al., 2015), are likely explained by factors other than sediment manipulation. These might reasonably include differences in the environmental conditions of the natural systems analyzed, in the diversity of the benthic microbes, and/or in the metabolism of the benthic prokaryotic assemblages.

In line with this, our results indicate that, independent of the approach used for sediment processing, the viral production rates we determined were significantly higher in the samples collected at the deepest water depth. This can be dependent upon the different environmental settings of the two stations, including a higher availability of trophic resources reported at the deeper station (Dell’Anno et al., 2015), which can support high viral production rates through enhanced prokaryotic heterotrophic metabolism.

### Different Radiotracer-Incorporation Methods to Assess Prokaryotic Production Rates and Effects of Sediment Dilution

Contextual assessment of viral and prokaryotic production rates is fundamental for a reliable evaluation of the role of viruses in benthic carbon cycling and in the overall microbial food web functioning (Middelboe and Glud, 2006; Danovaro et al., 2008a,b). To provide independent assessment of the impact of viruses on their prokaryotic hosts, in our study the rates of prokaryotic heterotrophic carbon production were determined by analysing different steps of cellular macromolecular anabolic pathways: DNA and protein synthesis, respectively, based on ^3^H-thymidine incorporation into the cellular genome, and on the incorporation of ^3^H-leucine into amino acids and peptides. To our knowledge, only van Duyl and Kop (1994) compared these two methods synoptically in previous studies on deep-sea samples, reporting similar results. However, these analyses included samples collected at depths up to 350 m, making ours the first comparison of such methods for deep-sea sediment samples retrieved from below 1000 m water depth. Our findings corroborate previous evidence of consistency observed between the two different methods, even at depth. Our findings also highlight that important sources of variability include the radiotracer selected, and the CF needed to transform the amount of carbon produced into a cell number (Danovaro, 2010 and references therein). In this regard, the CF commonly used (Danovaro, 2010; Danovaro et al., 2015), adopted also in the present study (i.e., 310 fg C μm^-3^, Fry, 1990), provided average contents of 23.5 ± 2.1 fgC per cell, in line with that usually assumed in marine microbiology studies (20 fgC per cell; Cho and Azam, 1990; Ducklow and Carlson, 1992). Moreover, our values are consistent with recent independent empirical evidences obtained from studies conducted on surface and subsurface sedimentary prokaryotic cells (21.5 ± 4.4 fgC per cell; Braun et al., 2016). These authors reported relatively low coefficients of variation for these experimental measures (ca. 20%), suggesting that this commonly assumed CF can provide reliable estimates.

It is well known that the production of new viruses correlates with the metabolism of prokaryotic host cells (Wommack and Colwell, 2000; Weinbauer, 2004; Danovaro et al., 2008b). Thus, potential changes in host metabolism due to sediment manipulation (homogenisation and/or slurring; Moriarty et al., 1991; Hansen et al., 2000) would be expected to influence also viral production rates. Our results show that, in both deep-sea stations, the values of prokaryotic C production rates did not differ between sediment samples incubated as intact whole cores or diluted, independent of the analytical procedure used for their determination (i.e., ^3^H-leucine or ^3^H-thymidine incorporation). As such, we can conclude that bias caused by a potential enhancement of prokaryotic metabolism following sediment manipulation can be ruled out, at least within the short time period of incubations required by the dilution-based approach (i.e., 12 h).

### Viral Production Rates by EFM and by the ^3^H-Thymidine Method

When compared with the methodology based on incorporation of radiotracers, the procedures based on viral counts by EFM have the advantage of a direct assessment of viral production rates without the use of CFs needed for the incorporation of radiolabeled substrates (Bettarel et al., 2006; Filippini et al., 2006). However, to further assess the reliability of different methods for determining viral production in surface deep-sea sediments, we included an independent approach, based on ^3^H-thymidine incorporation into viral genomes. This approach, previously applied only to seawater samples including deep-sea waters collected at the water-sediment interface (Steward et al., 1992, 1996; Noble and Steward, 2001; Helton et al., 2005; Danovaro et al., 2008b), has been applied here for the first time to the analysis of marine sediments. The ^3^H-thymidine method typically requires a CF to express the moles of radiotracer incorporated as viral production rates (Steward et al., 1992). The use of the sample-specific CFs empirically determined at 450 and 1900 m depth provided values of viral production consistent with those determined by EFM. Conversely, our study confirms that the use of the theoretical CF of 0.024 × 10^21^ viruses produced per mole of ^3^H-thymidine incorporated can underestimate the actual viral production rates (Noble and Steward, 2001) (**Figure 3B**). This is likely due to the fact that the theoretical CF does not take into account factors such as isotope dilution during DNA synthesis (Noble and Steward, 2001). Indeed, viral production rates determined using sample-specific CFs were in the range of those obtained with the empirical CFs previously determined from water column samples (Steward et al., 1992; Danovaro et al., 2008b) (**Figure 3B**), indicating that the assumption of the theoretical CF can underestimate viral production rates similarly in seawater and sediment samples (Noble and Steward, 2001; this study). Moreover, our data demonstrate that the use of standard CFs can overestimate or underestimate the virus production rates when compared with those determined using the sample-specific CFs (**Figure 3B**) obtained by intercalibration with EFM. These results suggest the need of determining the CF in each environmental setting for obtaining reliable estimates when we use the radiotracer method.

The fact that specific groups of benthic prokaryotes are known that do not incorporate ^3^H-thymidine into viral DNA (Gilmour et al., 1990), poses further questions on the general applicability of the ^3^H-thymidine approach in the assessment of viral production rates. These considerations, as well as those related to safety and waste disposal of radioactive materials, lead us to suggest microscopy-based approaches as more accurate and reliable compared to the radiotracer method. However, it has to be noted that also when using EFM, appropriate controls and experimental validation need to be included, such as optimization of virus and cell extraction from sediments, staining protocols, DNase treatments and counting under EFM (Dell’Anno et al., 2009; Danovaro, 2010; Danovaro and Middelboe, 2010). Moreover, proper and steady settings have to be maintained during incubations, including *in situ* temperature values and dark conditions, to avoid biases possibly originating by temperature-associated changes in microbial metabolism and/or by exposure of the sediments to light (Danovaro, 2010).

### Assessing the Impact of Viruses on Their Prokaryotic Host

**FIGURE 5:**
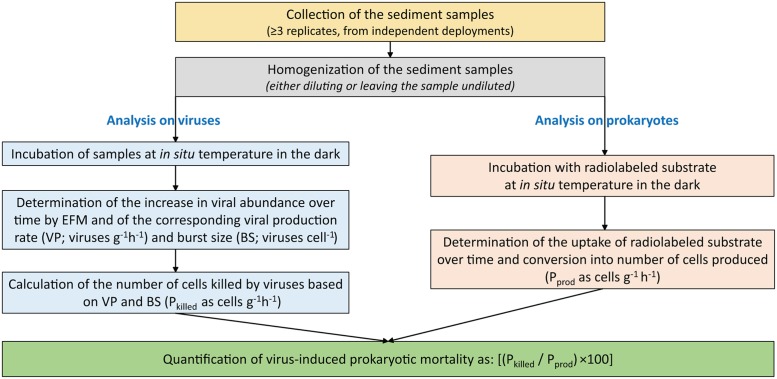
**Diagram for the quantification of the viral production in benthic ecosystems.** Reported is the flow chart of the different steps required for the analysis of viruses and prokaryotes and the impact of viruses on their hosts. P_killed_ is the number of prokaryotic cells killed by viruses; P_prod_ is the number of prokaryotic cells produced. Details are in the “Materials and Methods” section.

In the present study, we found a significantly higher impact of viruses (as virus-induced prokaryotic mortality) at the deeper station, coupled with faster turnover times of prokaryotic biomass. These results, although obtained at only two sampling depths, agree with the expectation of an increase of viral “predatory” pressure on benthic prokaryotes with increasing water column depth (Danovaro et al., 2008b), and with the known potential for viral lysis to stimulate host metabolism, thus accelerating biomass turnover (Danovaro et al., 2008b; Weitz et al., 2015). Additionally, the values of burst sizes we estimated are consistent with those previously reported for deep-sea sediments around the world, obtained using TEM and counting of visibly infected cells (average BS of 45 virus per lysed cell; Danovaro et al., 2008b). This suggests that EFM-based methods might be a reasonable opportunity if cost-benefit constrains preclude availability of more expensive equipments such as electron microscopes. This holds true considering the much higher uncertainty (coefficient of variation, 105–130%, fivefold to ninefold more variable than those determined by the EFM method) in the viral production rates (and hence, in the impacts of viruses on their prokaryotic hosts) determined by the ^3^H-thymidine method if not using sample-specific CFs. A flow chart synoptically showing the proposed different steps required for the analyses of viruses and prokaryotes when assessing the impact of viruses on their prokaryotic hosts is reported in **Figure 5.**

## Conclusion

Overall, the results reported in our study provide evidence of consistency between dilution-based and non-dilution based approaches for the determinations of viral and prokaryotic production rates in benthic ecosystems (Danovaro et al., 2008a,b; Dell’Anno et al., 2009, 2015). Indeed, our synoptic comparisons provide the first solid evidence that different approaches for sediment processing and for the assessment of viral and prokaryotic production rates can provide similar results. This suggests that it is possible to cross-compare findings obtained via independent studies using the various virus production methods presented here. Our results suggest that the determination of viral production by direct counting under EFM should be recommended as this approach is far less laborious and time consuming, is simpler, reliable and cost-effective, and does not require the use radioactive compounds.

## Author Contributions

RD, MM, and RN conceived the study. ER, AD, and CC carried out the laboratory work, data analysis, and interpretation. ER, AD, and CC wrote the paper with input and revisions from RD, RN, and MM. All authors contributed to discussing and reviewing the results and agreed to the final article content.

## Conflict of Interest Statement

The authors declare that the research was conducted in the absence of any commercial or financial relationships that could be construed as a potential conflict of interest.
